# Enhancing public sector employees ’ professional identity: A study using China’s Labor Force Survey data

**DOI:** 10.1371/journal.pone.0313603

**Published:** 2025-01-09

**Authors:** Xiaotong Ren, Aimin Zhu, Jiao Yu, Chunna Guo

**Affiliations:** 1 School of Management, Shenyang University of Technology, Shenyang, China; 2 School of Management, Shenyang Normal University, Shenyang, China; 3 School of Economics and Management, Inner Mongolia Normal University, Inner Mongolia, China; National University of Modern Languages, PAKISTAN

## Abstract

Public sector employees, as the crucial link between the party, government, and the general public, represent the frontline in safeguarding the interests of the people and providing services to them. They profoundly influence the implementation and execution of national policy directives. Using data from China’s Labor Force Survey and employing a combination of exploratory factor analysis and confirmatory factor analysis, this research constructs an evaluation index system for public sector employees’ Professional Identity. By dividing the evaluation index system into secondary indicators, and utilizing a structural equation standardization model, the study accurately determines the extent to which each level of indicators within the evaluation index system influences Professional Identity. This research provides pathway recommendations to enhance the Professional Identity of public sector employees, with the aim of mitigating occupational burnout and improving the quality of public services.

## Introduction

Civil servants, as a vital force within the national institutions, play a crucial role in the operation of the government and the provision of public services. Professional identity among civil servants, as a psychological state, reflects the degree of their identification with their profession. It encompasses various aspects such as professional identity, values, sense of professional responsibility, loyalty to the organization, and dedication to public affairs. In modern society, with ongoing social changes and increasing complexity in public administration, the study of civil servants’ professional identity has become particularly significant.

According to reports, 78.9% of grassroots civil servants experience varying degrees of occupational burnout [1]. This phenomenon cannot be ignored, as grassroots civil servants serve as a crucial link between the government and the general population, and their enthusiasm and work quality directly impact the implementation of national policies and reforms [2]. Positioned at the bottom of the hierarchical system, grassroots civil servants bear heavy work responsibilities, often facing a series of challenges due to factors such as limited resources and relatively low compensation, leading to the occurrence of professional burnout [3]. Firstly, they shoulder a multitude of tasks, facing prominent work pressures. The heavy workload has placed significant stress on some civil servants, leading to an intensified sense of psychological burnout. Secondly, the slow pace of promotion and limited career development opportunities for grassroots civil servants, coupled with the dilemma of encountering a "glass ceiling" in promotions, results in a stagnation of job levels over the years. This narrow promotion pathway undermines the motivation of grassroots civil servants [4]. Finally, inadequate motivation and unmet needs are also challenges faced by grassroots civil servants. The existing evaluation mechanisms and compensation and benefits systems suffer from insufficient scientific rigor and lack specificity, making it difficult to provide effective incentives for grassroots civil servants. This situation is detrimental to the enhancement of civil servants’ professional identity. Against this backdrop, there is a need to explore a suitable evaluation indicator system for civil servants and empirically test it. Researching motivational methods from a psychological perspective and alleviating occupational burnout among civil servants can contribute to effectively enhancing their professional identity. This, in turn, aims to improve the quality of public service delivery.

This study makes significant contributions in the following aspects: (1) Grounded in the ERG theory and other relevant frameworks, it introduces the research on professional identity into the field of civil service, expanding the scope of civil service studies. The study enriches the exploration of professional identity’s impact on subjects in the public sector, providing new perspectives and research directions for public administration theory. (2) Through empirical analysis, the study examines the influencing factors of civil servants’ professional identity. It establishes an evaluation index system for the sense of professional identity among civil servants, addressing the quantitative research gaps in current studies on civil servants’ professional identity. This research holds significant theoretical implications for the professional development and organizational management of civil servants.

## Literature review

### The occupational psychology of civil servants

In the past few decades of administrative reforms, there have been active or passive changes in the identity, perspectives, and performance of civil servants. Whether such changes will impact the social identity of civil servants is worth contemplating [5]. Martijn explored the value patterns guiding civil service work, summarizing four governance perspectives from previous literature and transforming them into a comprehensive set of values guiding civil servant actions [6]. Sloan analyzed emotional labor issues among public sector employees, further clarifying the relationship between emotional labor and worker well-being [7]. Cable conducted a comparative analysis of the impact of social interactions on the psychological health of civil servants in Japan, Finland, and the United Kingdom [8]. Nobue selected 3015 Japanese civil servants for an empirical analysis of the impact of workplace social capital on depression. The findings suggested that workplace social capital can mitigate the impact of work and family stress on civil servants’ depression [9].

The impact of COVID-19 on the psychological state of civil servants has also been a recent focus of research. Joelle, using the JD-R model, investigated the current crisis’s effect on the well-being of civil servants. They argue that, in the context of the COVID-19 pandemic, the cognitive perception of the role of civil servants directly influences their sense of well-being. Perceptions of job stress and life imbalance negatively affect well-being, while perceptions of self-efficacy, autonomy, and social support positively influence the attainment of well-being [10]. Azhar, in Indonesia, examined the factors influencing the work-from-home stress of civil servants during the COVID-19 pandemic. The study identified organizational changes and social support as significantly correlated factors with work stress and proposed relevant recommendations [11].

### Professional identity

Professional identity refers to an individual’s sense of belonging and identification with the profession they are engaged in, constituting a psychological concept. Early research primarily unfolded in the field of psychology, with Pumpianmindlin proposing a focus on enhancing professional identity in the training of psychologists, marking the beginning of research on professional identity across various professions [12]. Hendry, LB analyzed the professional identity of physical education teachers in marginal roles from a psychological perspective [13]. As research on professional identity deepened, the focus gradually expanded to various industries. For instance, David conducted interview data analysis using grounded theory, examining the professional identity of safety professionals from the perspective of their occupational roles. The study ultimately constructed a professional identity model consisting of experience, motivation, beliefs, and values [14]. Deneen, on the other hand, focused on female engineers as interview subjects and analyzed the impact of gender on the professional identity of engineers [15]. Stack conducted qualitative research on the professional identity issues of auditors, reviewing and summarizing literature on the formation of professional identity. The study proposed future research directions for the professional identity of auditors [16]. In addition, scholars have also undertaken research on the professional identity of librarians, proposing a conceptual model for the formation and development of librarian professional identity. The study explored the professional identity of librarians from a sociological perspective [17].

From an overall perspective, research on professional identity can be categorized into two main directions: studies on the professional identity of teachers and healthcare professionals. The investigations in these two domains serve as the foundation for research into the sense of identity in other professions. Firstly, there is an in-depth exploration of teachers’ professional identity, with academia delving into the factors that influence it. Androusou conducted an analysis of the professional experiences of Greek teachers, investigating the factors that affect the professional identity of teachers in Greece [18]. Furthermore, Kaurgo constructed a model for teacher professional identity, encompassing dimensions such as identity beliefs, emotional events, and identity negotiation. The aim is to analyze the role of teachers’ emotions in the formation of their professional identity [19].

Pnina further validated the beneficial facilitating role of on-site conflict simulation in fostering the professional identity of teachers at the Israeli College of Education [20]. Additionally, there has been a growing interest among researchers in exploring the professional identity of student teachers and education majors. These studies aim to investigate how emphasizing the cultivation of professional identity from the initial stages of a teaching career may have an impact [21, 22]. Another significant direction in research is the exploration of the professional identity of healthcare workers. Some scholars categorize the professional identity of nurses into three types: self, role, and nursing practice situations. Building upon this framework, Philippa delves into whether nurses’ perceptions of their professional identity and influencing factors undergo changes over the course of their practice [23].

On another note, Dogan investigated the impact of a professional identity development program on the job satisfaction and levels of professional burnout among registered nurses [24]. Meanwhile, Szulik conducted qualitative research exploring the influence of professional identity on physician well-being. The study suggests that enhancing physicians’ perceived happiness can be achieved through the development of a strong professional identity, while also emphasizing the need to address potential negative factors such as commitment and self-concept [25]. Additionally, Cullum examined how the practice community model guides the development of medical students’ professional identity in the context of the COVID-19 environment. The study provides recommendations for educators and institutions to consider professional identity in future planning, addressing the challenges posed by the current situation [26].

### ERG theory

In 1969, Alderfer proposed the ERG (Existence, Relatedness, and Growth) Needs Theory, emphasizing that individuals have different levels of needs at various stages of life and work. This theory, based on Maslow’s Hierarchy of Needs, modifies and simplifies the five needs categories, proposing three core needs: Existence, Relatedness, and Growth. ERG theory posits that various human needs are flexible and coexist, constantly strengthening. In this study, when defining dimensions for the assessment of professional identity, reference will be made to the three core needs of ERG theory. The conditions of existence, relatedness, and growth among civil servants directly impact their service quality and professional identity. Furthermore, ERG theory serves as theoretical support for subsequent strategies aimed at enhancing the professional identity of civil servants.

### Summarize

After careful review and analysis, we have found that the academic community has made substantial progress in the study of the psychological aspects and professional identity of civil servants, with profound insights and extensive coverage. Despite the relatively abundant research in the field of professional identity, studies specifically focusing on civil servants are scarce. Therefore, upon reviewing existing research, we have identified the following observations: (1) The majority of existing studies predominantly emphasize the surface-level sense of professional identity, with a primary focus on individuals’ emotional attitudes towards the organization and profession. This limited perspective hinders a comprehensive understanding of the essence of civil servants’ professional identity. Consequently, there is a lack of scientific basis for strategies guiding managers on how to effectively lead and enhance the professional identity of civil servants in a targeted manner. (2) Research on the professional identity of civil servants has direct practical significance in enhancing public administration efficiency and optimizing the level of public services. It contributes to a deeper theoretical understanding of human resources management in the public service sector. By comprehending the mechanisms and influencing factors behind the formation of civil servants’ professional identity, more effective talent development and motivation strategies can be devised for the public sector. This, in turn, facilitates the modernization of management practices within public service institutions.

## Methods

### Data source and processing

The data analysis in this study is derived from the China Labor Dynamics Survey (CLDS). The CLDS survey covered a nationwide scope, including 26 provinces, 14,226 households, and 21,086 individuals, exhibiting a highly representative sample. The CLDS main questionnaire consists of three sections: village, individual, and household. In this study on the professional identity of public sector employees, it primarily involves questions from the individual and household questionnaires related to job types and job satisfaction. As a result, data from the individual and household questionnaires were merged and processed. The study concentrates on public sector employees. Consistent with the definition of the public sector within the Chinese academic framework, cases categorized under ’party, government, military agencies’ and ’public institutions’ were retained in the dataset, resulting in a final sample size of 1,153. The essential details of the processed sample are presented in Table 1.

**Table 1:** Basic information of the sample.

Heading	Quantity	Percentage Composition(%)
Gender		
Male	666	57.8
Female	487	44.2
Village Type		
Rural	226	19.6
Urban	927	80.4
Household Registration Type		
Agricultural	233	20.2
Non-Agricultural	920	79.8
Political Affiliation		
Party Member	411	35.6
Masses	742	64.4
Ethnicity		
Han	588	51
Other	585	49
Educational Background		
Associate Degree and Below	462	40.1
Bachelor’s Degree and Above	691	59.9
Nature of the Unit		
Party, Government, and Military	273	23.7
Public Institution	880	76.3

Data from the respondents reveals that the sample is skewed toward males, accounting for 57.8% of the total. There is a significant disparity in the distribution of village types, with urban areas making up 80.4%, surpassing rural areas by a margin of 60 percentage points. This substantial gap also extends to the nature of household registration, with agricultural and non-agricultural household registrations differing by nearly 60 percentage points. From this, it can be inferred that the types of villages (neighborhood committees) and non-agricultural household registrations have a significant impact on public sector employee recruitment, indicating a certain urban-rural divide.

Out of the 1,153 civil servants, 742 are members of the Communist Party, accounting for 64.4% of the sample. The ethnic distribution of the sample is reasonably balanced, differing by only 3 samples. In terms of education, 60% hold a bachelor’s degree or higher, aligning with the current trends of ’civil service exam popularity’ and ’educational attainment competition.’ Among these public sector samples, there are 273 cases from party, government, and military agencies, representing 23.7%, while public institutions account for 76.3% with 880 cases.

### Statistical analysis

In this phase, the analysis was conducted using SPSS software. Initially, relevant factors for Professional Identity, such as income, promotion opportunities, and working hours, were selected from the China Labor Dynamics Survey database, drawing on existing research in academia. Subsequently, the reliability and validity of these selected items were examined to assess the overall structural integrity. Following this, an exploratory factor analysis was employed on the tested data to determine the hierarchical dimensions of the evaluation index system, establishing the framework for evaluating the Professional Identity of public sector employees. Finally, utilizing the identified hierarchical dimensions, a structural equation model was constructed to validate the rationality of the developed index system.

## Results

### Reliability and validity analysis

Reliability analysis is a method used to examine the consistency of results from multiple tests of the same method, also known as a reliability test, according to the standards of scientific investigation. The higher the consistency of test results and the smaller the errors, the greater the reliability. The data utilized in this study are derived from the Chinese Labor Force Survey Database, with a certain level of assurance regarding the reliability of questionnaire items. However, after referring to the relevant literature, select the specific items in the questionnaire that have an impact on professional identity (Table 2) [27–29], an overall reliability analysis of the chosen items is necessary to ensure the reliability of the research results.

**Table 2:** Multiple choice item list.

item	question
I7.3.1income	Please evaluate your job income
I7.3.2job security	Please evaluate your job security
I7.3.3work environment	Please evaluate your work environment
I7.3.4working hours	Please evaluate your working hours
I7.3.5promotion opportunities	Please evaluate your job promotion opportunities
I7.3.6job interest	Please evaluate the interest of your job
I7.3.7work collaborator	Please evaluate your work collaborators
I7.3.8utilization of abilities and skills	Please evaluate the use of your skills and abilities at work
I7.3.9gaining respect	Please evaluate the level of respect shown to you by others
I7.3.10expressing opinions	Please evaluate the opportunities for expressing opinions
I7.6.1well-being	Overall, do you feel that your life is happy
I7.6.2life satisfaction	Overall, are you satisfied with your life situation
I7.6.3economic satisfaction	Overall, are you satisfied with your family’s financial situation

Using the obtained 13 items as variables, a reliability analysis was conducted to assess the structural validity. After a preliminary categorization based on the nature of the questions, the Cronbach’s coefficients for each dimension were calculated, as presented in Table 3. It is evident that the Cronbach’s α values for the dimensions of objective conditions, subjective perceptions, and external environment are 0.836, 0.853, and 0.837, respectively, all exceeding 0.7. This indicates a robust correlation and internal consistency among the factor variables within each dimension.

**Table 3:** Reliability analysis.

Measurement Dimensions	Survey Items	Mean	Standard Deviation	Cronbach’s α
Objective Conditions	A1 Income	2.798	0.9735	0.836
	A2 Working Environment	2.309	0.8038	
	A3 Promotion Opportunities	2.351	0.7880	
	A4 Job Security	2.416	0.8252	
	A5 Working Hours	2.857	0.8393	
	A6 Job Interest	2.690	0.8746	
	A7 Co-workers	2.303	0.7445	
Subjective Perceptions	B1 Respect	2.318	0.7060	0.853
	B2 Ability and Skills	2.344	0.7024	
	B3 Expressing Opinions	2.447	0.7992	
External Environment	C1 Life Satisfaction	4.017	0.8056	0.837
	C2 Life Happiness	3.951	0.8397	
	C3 Economic Satisfaction	3.509	0.9887	

Valid Cases: 1153

### Validity analysis

The subsequent phase entails validating the data from two angles: content validity and structural validity. Regarding content validity, the items scrutinized in this study originate from the China Labor Force Survey database. These items have undergone rigorous evaluation by expert scholars, affirming their robust content validity. Consequently, attention turns to the structural validity assessment of the chosen multiple-choice items. The methodology utilized for testing structural validity adheres to established research practices and incorporates factor analysis.

The evaluation of data structural validity through the method of factor analysis primarily relies on the numerical values of KMO (Kaiser-Meyer-Olkin) and Bartlett’s test. Qualified sample data should meet three criteria: firstly, the KMO value should exceed 0.7, and the closer the obtained value is to 1, the stronger the correlation among variables; secondly, the sample’s chi-square value should be less than 0.05, indicating significant differences; thirdly, the presence of common factors is essential [30–33]. After analyzing the data, Table 4 was generated, with a KMO value of 0.883 and a chi-square value of 7417.385 for Bartlett’s test, which is significant at the p < 0.01 level. This indicates that the data is suitable for factor analysis. Previous research suggests that factors with cumulative explained variance above 60% are considered reliable. The total residual variance of the sample data is presented in Table 5, revealing the presence of three common factors with eigenvalues greater than 1 and a cumulative explained variance of 66.135%. Therefore, the selected combination of items exhibits good structural validity [34].

**Table 4:** KMO and Bartlett’s sphericity test.

The KMO sampling adequacy measure.		0.883
Bartlett’s sphericity test	Approximate chi-square	7417.385
	Degrees of freedom	78
	Significance	0.000

**Table 5:** Cumulative explained variance.

	Initial Eigenvalues			Sum of Squared Loadings			Rotated Sum of Squared Loadings		
	Total	Variance ratio	Cumulative	Total	Vari ratio	Cumulative	Total	Vari ratio	Cumulative
1	5.568	42.832	42.832	5.568	42.832	42.832	3.158	24.296	24.296
2	1.988	15.295	58.127	1.988	15.295	58.127	3.049	23.453	47.749
3	1.041	8.008	66.135	1.041	8.008	66.135	2.390	18.386	66.135
4	0.809	6.220	72.356						
5	0.605	4.652	77.008						
6	0.521	4.008	81.015						
7	0.497	3.827	84.842						
8	0.412	3.168	88.011						
9	0.397	3.057	91.068						
10	0.375	2.887	93.955						
11	0.307	2.365	96.320						
12	0.270	2.078	98.397						
13	0.208	1.603	100.000						

Extraction Method: Principal Axis Factoring

### Cumulative explained variance

Utilizing exploratory factor analysis to discern shared factors among the items and assigning them as secondary indicators within the evaluation index system. Subsequent to the analysis, adjustments and exclusions of items from the initial categorization were made, with the retained items serving as tertiary indicators. This process culminated in the establishment of the ultimate evaluation index system for assessing the professional identity of public sector employees.

**Fig 1:**
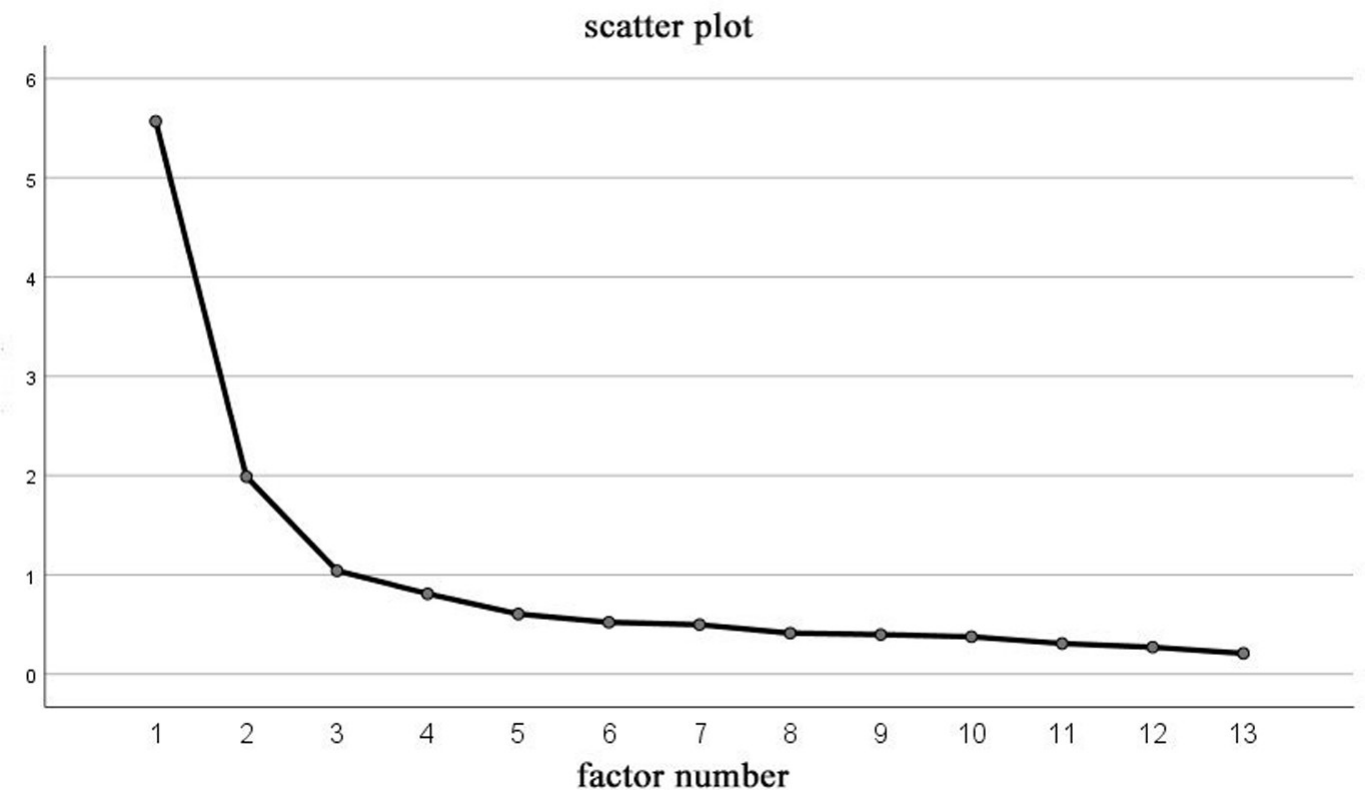
Scree plot for factor extraction.

The method chosen to determine the number of factors is the principal axis factor method, which is more suitable for exploring the data structure. After extracting the factors, common factors were retained based on the criterion of eigenvalues greater than 1 [35]. As shown in Table 4, there are 3 factors with eigenvalues greater than 1, indicating that selecting three common factors is a reasonable structure for the sample data. The scree plot (Fig 1) shows an inflection point at 3, and the curve becomes flat after 5, suggesting that the number of common factors should be between 3 and 5. Combining eigenvalues and the scree plot, the determined number of common factors is 3.

After determining the number of factors, factor rotation was performed to derive factor loadings for each indicator, thereby delineating the structure of the indicator system. Two prevalent methods for factor rotation exist: orthogonal rotation and oblique rotation. Some scholars contend that oblique rotation offers broader insights and more comprehensive information compared to orthogonal rotation. Consequently, oblique rotation is advocated over orthogonal rotation [36, 37].

The results of oblique rotation for the sample data are presented in Table 6. First, items with factor loadings less than 0.4 were removed, i.e., item A6 was deleted. Second, adjustments were made to the dimension induction based on the rotated factor loadings. According to the factor loadings in Table 5, the results of the factor analysis are consistent with the previous dimension division, indicating that no structural changes are needed.

**Table 6:** Factor loading table.

	factor		
	1	2	3
A1	-0.157	-0.175	0.708
A4	0.126	0.059	0.665
A2	0.119	0.071	0.756
A5	0.190	0.081	0.582
A3	0.125	-0.035	0.504
A6	0.331	-0.058	0.370
B3	0.687	-0.015	0.082
B2	0.805	-0.023	-0.022
B1	0.778	-0.057	0.031
B4	0.569	-0.066	0.219
C1	-0.133	0.826	0.090
C2	-0.065	0.936	0.070
C3	0.107	0.624	-0.233

Extraction Method: Principal Axis Factoring.

Rotation Method: Kaiser Normalization Oblimin.

a. Convergence was achieved after 17 iterations of rotation.

Based on Table 6, it can be observed that Factor 1 includes B1 Respect, B2 Ability and Skill Usage, B3 Work Collaborators, and B4 Opinion Expression. Therefore, this factor is named Subjective Mental Perception. Factor 2 comprises C1 Life Satisfaction, C2 Life Happiness, and C3 Economic Satisfaction, and is named External Overall Environment. Factor 3 includes A1 Income from Work, A2 Work Environment, A3 Opportunities for Work Promotion, A4 Job Security, and A5 Working Hours, and is named Objective Working Conditions. After arranging the indicators in a logical order, the evaluation index system for the Professional Identity of public sector employees is obtained (**Table 7**).

**Table 7:** Performance evaluation index system of public sector employees’ professional identity.

Primary Indicator	Secondary Indicator	Secondary Indicator
Professional Identity	Objective Conditions	Income
		Working Environment
		Promotion Opportunities
		Job Security
		Working Hours
	Subjective Experience	Respect
		Competence and Skill Utilization
		Work Collaboration
		Expression of Opinions
	External Environment	Life Satisfaction
		Happiness in Life
		Economic Satisfaction

### Confirmatory factor analysis

Following the initial establishment of the evaluation indicator system for civil servants’ Professional identity through exploratory factor analysis, this study employed the structural equation model (SEM) method to validate the rationality of the indicator system [38, 39]. This approach involves constructing a model based on the various dimensions of the evaluation indicator system and inputting sample data into the model. The feasibility of the model is assessed by calculating the results, serving as the basis for testing the rationality of the indicator system.

**Fig 2:**
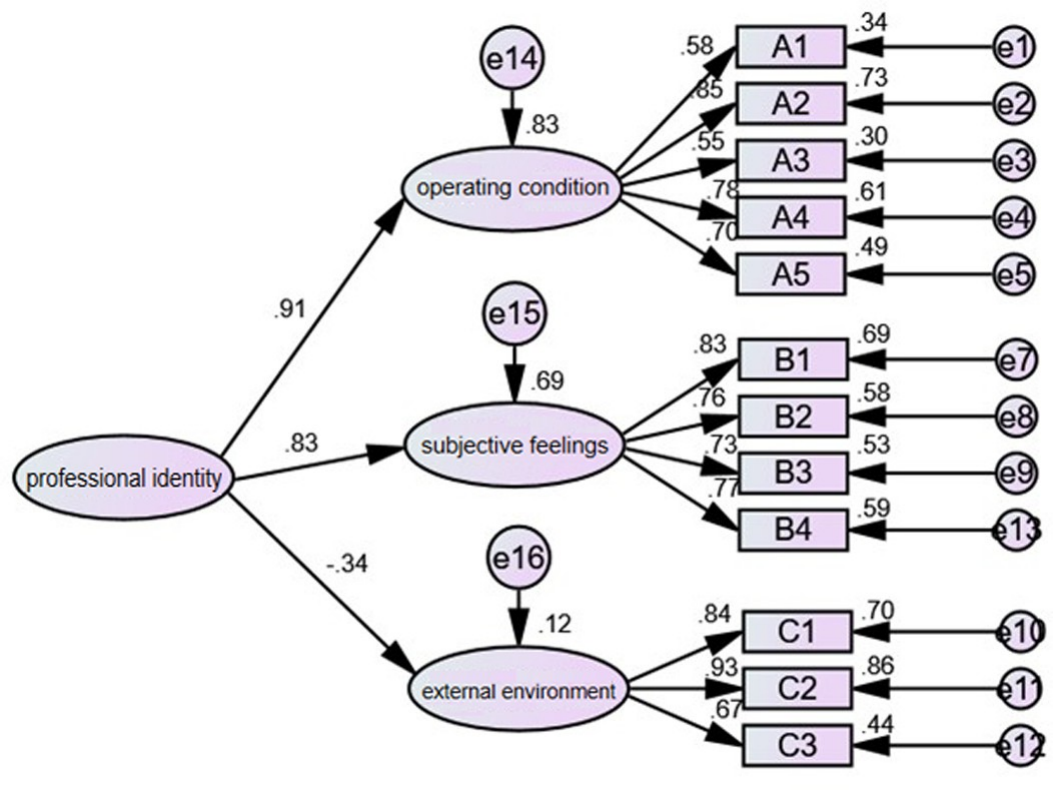
Structural equation model of public sector employee professional identity.

Structural equation model based on the dimensions of the public sector employee Professional Identity evaluation index system is presented in Fig 2. The figure displays the standardized results of confirmatory factor analysis, where the factor loading for each third-level dimension is consistently above 0.6, ranging from 0.55 to 0.93, indicating a reasonable outcome.

The results of the validity analysis of the model structure are presented in Table 8. The CR (Composite Reliability) values for objective conditions, subjective experiences, and external environment are 0.824, 0.855, and 0.854, respectively, all exceeding the standard of 0.8. The AVE (Average Variance Extracted) values are 0.492, 0.597, and 0.668. While the AVE value for objective conditions is slightly below the general standard of 0.5, considering the large sample size, it falls within an acceptable range. Furthermore, upon comparing the absolute values of factor correlation coefficients with the square root of the AVE for each factor, no coefficients were found to exceed the square root, indicating that the model exhibits good discriminant validity.

**Table 8:** Structural equation model validity and reliability test.

	Objective Conditions	Subjective Experiences	External Environment	Convergent Validity AVE	Composite Reliability CR
Objective Conditions	0.492			0.492	0.824
Subjective Experiences	0.757	0.597		0.597	0.855
External Environment	-0.313	-0.286	0.668	0.668	0.855
Square Root of AVE	0.701	0.773	0.817		

The fit indices for the initial model are presented in Table 9. Analysis of Table 9 reveals that the model demonstrates a favorable fit, with the majority of indices falling within an acceptable range. This indicates that the current measurement model has initially met the criteria for excellence, signifying the viability of the Professional Identity evaluation measurement model developed in this study. It validates the rationality of the structure of the evaluation index system for professional identity among public sector employees.

**Table 9:** Structural equation model fit index.

Exponential Type	numerical value	criteria
RMSEA	0.085	Good<0.1, very good<0.05, excellent<0.01
CFI	0.938	normal range:0.7–0.9, good>0.9, very good>0.95
NFI	0.931	normal range:0.7–0.9, good>0.9, very good>0.95
IFI	0.938	normal range:0.7–0.9, good>0.9, very good>0.95
RFI	0.91	normal range:0.7–0.9, good>0.9, very good>0.95
TLI	0.919	normal range:0.7–0.9, good>0.9, very good>0.95

## Discussion

This study utilized data from the China Labor Force Survey, focusing on samples of public sector employees for analysis. Initially, relevant items related to Professional Identity were selected from the survey questionnaire to form the primary dimensions. These dimensions were then condensed based on correlation, and exploratory factor analysis was employed to extract common factors as secondary indicators for the evaluation system. Naming and initial adjustments and deletions of dimensions were carried out based on eigenvalues and factor loadings, resulting in tertiary indicators and the preliminary formation of the evaluation system. Subsequently, confirmatory factor analysis was used to construct the corresponding structural equation model. The data were input into the equation for an analysis of the model fit, assessing the degree of fit as an indicator of the model’s appropriateness. Based on the results of the fit analysis, the structural rationality of the evaluation system was verified, leading to the final establishment of the public sector employees’ Professional Identity Evaluation Indicator System. This system comprises three secondary indicators—Objective Working Conditions, Subjective Mental Perception, and External Overall Environment—each containing 12 tertiary indicators.

In the standardized structural equation model, the path coefficients to some extent indicate the significance of each indicator for the model. Therefore, the path coefficients of the public sector employee Professional Identity evaluation model can be considered as a basis for assessing the impact of each level of indicators on Professional Identity. This provides a foundation for formulating strategies for public sector employee Professional Identity in subsequent research.

Among the three secondary indicators, the most influential factor on public sector employee Professional Identity is the objective working conditions, with a high path coefficient of 0.94. This indicates that this dimension has an extremely significant impact on Professional Identity. Under objective working conditions, there are five tertiary indicators, including work income, environment, promotion opportunities, work safety, and work hours. In descending order of importance, these are ranked as work safety, work hours, work environment, work income, and promotion opportunities.

The indicator of subjective mental perception also has a significant impact, with a coefficient of 0.84. This secondary indicator comprises sub-dimensions such as respect, ability and skill utilization, work collaborators, and expression of opinions, with respect being the most influential. The path coefficient for the external overall environment is -0.35, indicating a reverse impact on Professional Identity. The sub-dimensions include life satisfaction, happiness, and economic satisfaction. This suggests that when public sector employees experience higher levels of happiness and satisfaction in society, it has a reverse impact on their own Professional Identity.

## Conclusion

Based on the data analysis results, the impact of two secondary indicators, Objective Working Conditions and Subjective Mental Perception, on Professional Identity warrants careful examination. The exclusion of the External Environment indicator is justified for two reasons. First, the negative path coefficient indicates that this indicator exerts an inverse effect on overall Professional Identity. Second, the sub-dimensions of this indicator—Life Satisfaction, Life Happiness, and Economic Satisfaction—are significantly influenced by macro-environmental factors. The positive impact of these sub-dimensions on Professional Identity is considerably less pronounced compared to Objective Working Conditions and Subjective Mental Perception. Therefore, the discussion on enhancing the Professional Identity of public sector employees will focus on the dimensions of Objective Working Conditions and Subjective Mental Perception.

### 1. Objective working conditions

In the context of objective working conditions, the most prominent influence on professional identity stems from the work environment. The work environment can be categorized into static and dynamic environments based on various criteria. The primary characteristic of the work environment lies in its dynamism, prompting employees to proactively enhance their work in order to adapt to the dynamic surroundings [40]. The overall work environment also influences employees’ work engagement. When environmental conditions align with employees’ basic psychological needs, their work motivation is stimulated, leading to an increase in the level of work engagement. Consequently, subjective feelings of happiness and fulfillment are also enhanced [41].

The impact of working hours on professional identity within the objective working conditions is highly significant. This is especially pronounced in the fast-paced lifestyle where the boundary between work and personal life becomes blurred, exacerbating the challenges related to the control of working hours. Excessive working hours not only increase the number of stimuli employees face, intensifying health risks, but also contribute to the normalization of overwork. This not only exacerbates physical health issues among employees but also diminishes occupational happiness and job satisfaction, exacerbating issues related to occupational burnout [42, 43]. Therefore, it is essential to schedule the working hours of public sector employees reasonably, maintaining them within the reasonable range of 40 hours per week. This ensures the well-being of public sector employees, enhances work performance, increases professional identity, and ultimately provides better services to the people.

The influence of salary and income on professional identity is also applicable to civil servants [44]. The compensation system for civil servants still faces underlying issues, such as the lack of a rationalized mechanism for salary growth, particularly a core mechanism centered around a general salary adjustment. The complexity of this issue lies in the analysis of influencing factors and the determination of the frequency of general salary adjustments. Simultaneously, it is crucial to adjust the compensation levels reasonably based on the actual job responsibilities and difficulty levels of civil servants, ensuring they receive appropriate remuneration. Additionally, considerations should be given to factors such as geographical location and years of service, leading to the implementation of a structurally differentiated compensation system for civil servants.

### 2. Subjective mental perception

The subjective mental perception index has a significant impact on professional identity, with a coefficient of 0.84. The importance ranking of the three-level indicators is as follows: respect, expression of opinions, ability and skill utilization, and work collaborators. Respect, a rarely explored dimension in existing studies on public sector employees, has been indicated by research to be a crucial influencing factor on job satisfaction [45]. When department employees feel pride and respect, their intrinsic motivation is stimulated, a sense of responsibility is greatly enhanced, and they tend to consider the organization more, increasing work engagement [46]. Conversely, when professional respect is lacking, employees may experience emotional exhaustion and show signs of burnout and a desire to leave [47]. To enhance the professional identity of public sector employees, respect is a crucial factor that needs to be addressed. Future research should focus on how to increase the perception of respect in the practical work of public sector employees.

In the workplace, collaborators refer to individuals with whom civil servants work, including colleagues, superiors, and subordinates, to accomplish tasks. The attitudes, abilities, and behaviors of these collaborators significantly impact the professional identity of civil servants. Public sector organizations can enhance collaboration by implementing measures such as providing fair evaluations and rewards and fostering a collaborative culture. These efforts aim to strengthen the professional identity of civil servants.

The expression of opinions and the use of skills and abilities tend to fulfill employees’ need to be valued in their job positions. This reflects the need for self-realization, driving employees to meet the demands of their work. When these aspects are not satisfied in the workplace, negative emotions may arise, leading to a decline in employees’ self-awareness. The sense of achievement derived from work gradually diminishes, resulting in decreased job satisfaction and the emergence of occupational burnout issues [48]. Occupational burnout is a prevalent issue within the current public sector system, and emotional exhaustion is a central element of occupational burnout [49]. Therefore, when considering the psychological issues of civil servants, it is essential to focus on their self-realization needs. Addressing these needs at the root can control the occurrence of burnout, enhance their sense of professional identity, increase work engagement, better provide public services, and ultimately achieve the goal of improving government satisfaction.

### Research gaps and future prospects

This article has made a significant contribution to the construction of a professional identity evaluation index system for civil servants. While the methods employed in the article assist in determining the evaluation index system and the weights of each indicator, there is a lack of analysis and demonstration regarding the causal relationships between the indicators. The interplay among the indicators and their impact on professional identity is a crucial consideration in constructing an evaluation index system. Future efforts should focus on strengthening the analysis and research of the causal relationships among the indicators. In addition, the data used in this study is cross-section data, which is not continuous. Future studies should pay attention to using multi-year data for analysis to verify the analysis results of this paper.
